# Modulation of glycine receptor single-channel conductance by intracellular phosphorylation

**DOI:** 10.1038/s41598-020-61677-w

**Published:** 2020-03-16

**Authors:** Gustavo Moraga-Cid, Victoria P. San Martín, Cesar O. Lara, Braulio Muñoz, Ana M. Marileo, Anggelo Sazo, Carola Muñoz-Montesino, Jorge Fuentealba, Patricio A. Castro, Leonardo Guzmán, Carlos F. Burgos, Hanns U. Zeilhofer, Luis G. Aguayo, Pierre-Jean Corringer, Gonzalo E. Yévenes

**Affiliations:** 1 Department of Physiology, Faculty of Biological Sciences, University of Concepción, Concepción, Chile; 2 Department of Pharmacology and Toxicology, Indiana University School of Medicine, Indianapolis, IN 46202 USA; 3 Institute of Pharmacology and Toxicology, University of Zurich, Winterthurerstrasse 190, CH-8057 Zürich, Switzerland; 4 Institute of Pharmaceutical Sciences, Swiss Federal Institute of Technology (ETH) Zurich, Vladimir-Prelog-Weg 1-5/10, CH-8090 Zurich, Switzerland; 5 Channel-Receptors Unit, Institute Pasteur, UMR 3571 CNRS, 75015 Paris, France

**Keywords:** Biophysics, Neuroscience

## Abstract

Glycine receptors (GlyRs) are anion-permeable pentameric ligand-gated ion channels (pLGICs). The GlyR activation is critical for the control of key neurophysiological functions, such as motor coordination, respiratory control, muscle tone and pain processing. The relevance of the GlyR function is further highlighted by the presence of abnormal glycinergic inhibition in many pathophysiological states, such as hyperekplexia, epilepsy, autism and chronic pain. In this context, previous studies have shown that the functional inhibition of  GlyRs containing the α3 subunit is a pivotal mechanism of pain hypersensitivity. This pathway involves the activation of EP2 receptors and the subsequent PKA-dependent phosphorylation of α3GlyRs within the intracellular domain (ICD), which decrease the GlyR-associated currents and enhance neuronal excitability. Despite the importance of this mechanism of glycinergic dis-inhibition associated with dysfunctional α3GlyRs, our current understanding of the molecular events involved is limited. Here, we report that the activation of PKA signaling pathway decreases the unitary conductance of α3GlyRs. We show in addition that the substitution of the PKA-targeted serine with a negatively charged residue within the ICD of α3GlyRs and of chimeric receptors combining bacterial GLIC and α3GlyR was sufficient to generate receptors with reduced conductance. Thus, our findings reveal a potential biophysical mechanism of glycinergic dis-inhibition and suggest that post-translational modifications of the ICD, such as phosphorylation, may shape the conductance of other pLGICs.

## Introduction

Glycine receptors (GlyRs) belong to the pentameric ligand-gated ion channel (pLGIC) family. GlyRs are anion-permeable channels, allowing the fast influx of chloride and the control of neuronal excitability. An individual GlyR subunit is composed by an extracellular domain (ECD), four transmembrane domains (TM1–4) and an intracellular domain between the TM3 and TM4 domains (ICD)^1–4^. To date, a single β subunit and four α subunits (α1–4) has been described. The α subunits share a high degree of sequence identity (≈75%). Nevertheless, they exhibit important differences in their biophysical and pharmacological properties as well as in their distribution along the CNS^1,3,4^.

In the mammalian CNS, GlyR activity critically controls neurophysiological functions such as motor coordination, respiratory control, muscle tone, as well as pain processing^2,3,5–13^. The importance of glycinergic inhibition was first recognized in studies using the GlyR antagonist strychnine^14,15^. Later, genetic studies found that mutations in the GlyR α1 and β genes are linked to hyperekplexia in humans^16^. More recent evidence has shown that specific GlyR subunits may play key roles in several diseases. For example, while the α1 subunit has been linked to tumorigenesis and alcohol intoxication^17,18^, mutations in the α2 subunit have been linked to autism^19^. Alterations in the RNA processing of α3 subunits generates hyperactive receptors, which have been related with epilepsy^20,21^. In addition, the functional inhibition of spinal dorsal horn α3GlyRs has been shown to be a critical mechanism in inflammation-induced pain hypersensitivity^2,22^. Furthermore, α3GlyRs have also been implicated in the modulation of respiratory rhythms^23^. Collectively, these studies indicate that the glycinergic system may be a promising target for future drug development.

The importance of glycinergic inhibition in chronic pain has been characterized in the superficial dorsal horn^22^. The proposed mechanism of α3GlyR-dependent pain sensitization involves the loss of glycinergic inhibition following the activation of neuronal EP2 receptors (EP2-R) by prostaglandin E2 (PGE2)^22,24,25^. EP2-R stimulation increases cAMP and subsequently promote PKA-dependent phosphorylation of the α3GlyR on the S346 residue within the ICD, decreasing the amplitude of glycinergic currents and enhancing the excitability of dorsal horn excitatory neurons. Interestingly, the relevance of α3GlyRs in pain sensitization has been highlighted by the recent characterization of allosteric modulators targeting GlyRs^26–29^. These reports have shown that compounds potentiating α3GlyR activity are able to reduce chronic pain symptoms in rodents. These results confirm the key role of α3-containing GlyRs in the analgesic effects of such modulators. However, our current understanding of the molecular events underlying the functional α3GlyR inhibition by PKA-mediated phosphorylation is still very limited.

Here we report that the activation of PKA signaling pathway decreases the unitary conductance of α3GlyR. In addition, we show that the substitution of the S346 amino acid with a negatively charged residue generate ion channels with lower conductance. Our findings propose an undescribed biophysical framework to understand a mechanism underlying neuronal dis-inhibition. In a broader context, our data suggest that dynamic modifications of the ICD chemical composition shape the conductance of pLGICs.

## Results

### Functional inhibition of α3GlyRs by PKA-mediated activation

**Figure 1:**
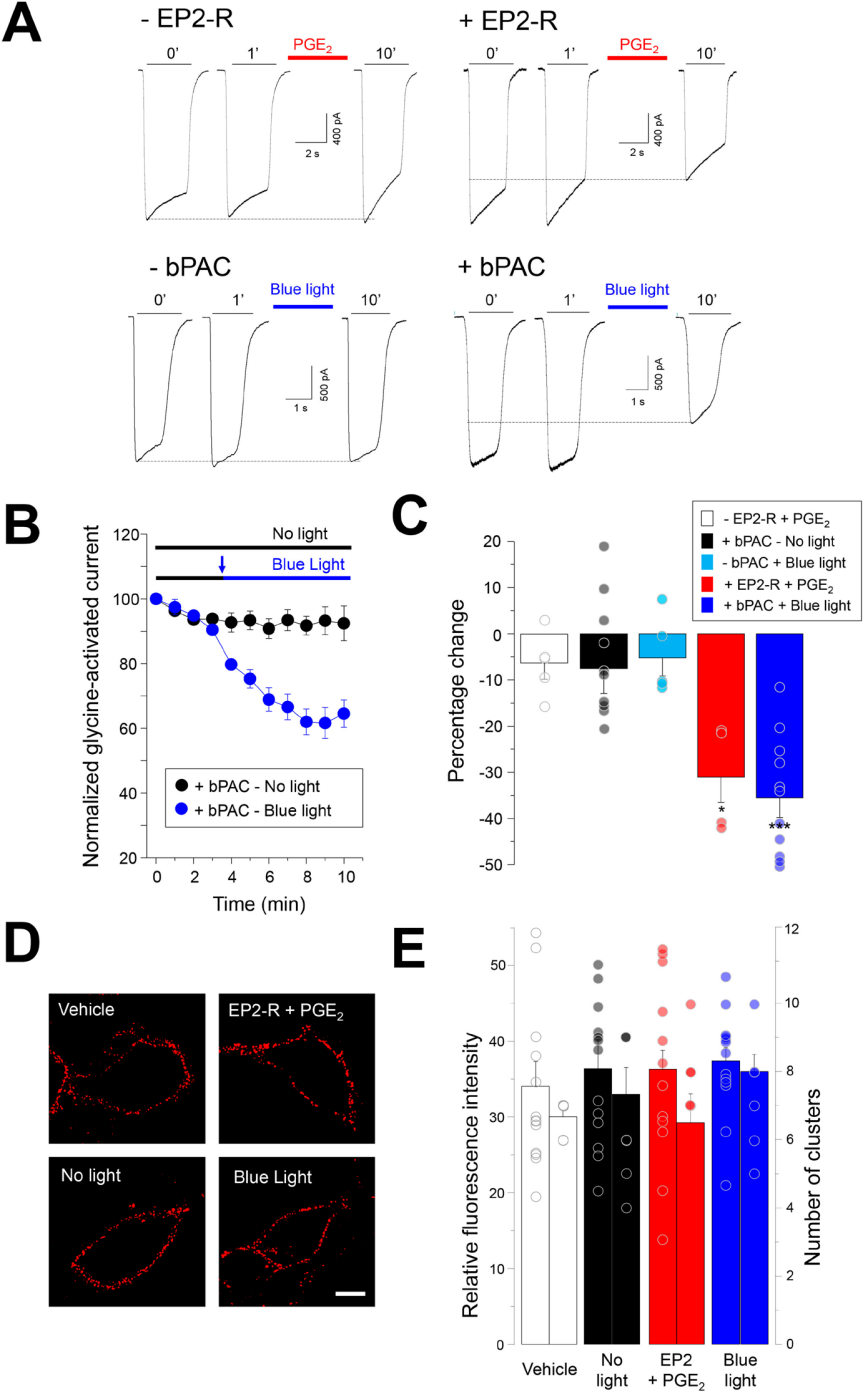
Functional inhibition of wild-type α3GlyRs by cAMP signaling. (**A**) Current traces from cells expressing α3GlyRs alone or together with EP2 receptors or with bPAC. Sample traces were obtained after 0, 1 and 10 minutes (‘) of recording. (**B**) Time course of glycine-evoked currents (500 μM) in the absence and presence of blue light in cells co-expressing α3GlyRs and bPAC. (**C**) Summary of the glycine-activated current inhibition elicited by EP2 receptor activation with PGE_2_ (n = 4, red) or by bPAC stimulation with blue light (n = 11, blue) after 10 minutes of recording. Control conditions (PGE_2_ without the expression of EP2 receptors (n = 5, white), +bPAC under no light conditions (n = 10, black) and cells stimulated with blue light without bPAC expressed (n = 5, cyan) are also shown. Differences were significant between the groups treated with PGE_2_ (*P < 0.05) and between the groups expressing bPAC (***P < 0.001). ANOVA followed by Bonferroni post-hoc test, F(4, 34) = 8.99. (**D**) Plasma membrane expression of α3GlyRs after EP2 receptor activation or after bPAC stimulation with blue light. Calibration bar, 5 μm. (**E**) The graph shows the quantification of the receptor-associated signals (left) and number of receptor clusters (right). Differences were not significant, (Fluorescence intensity, F(3, 51) = 0.25; number of receptor clusters, F(3, 31) = 1.1). Relative fluorescence intensity: control (vehicle), n = 13; no light, n = 13; EP2 + PGE2, n = 12; blue light, n = 14. Number of clusters: control (vehicle), n = 8; no light, n = 8; EP2 + PGE2, n = 8; blue light, n = 8.

We first assessed the effects of EP2-R activation on the glycine-activated currents through recombinant α3GlyRs expressed in HEK293 cells. As previously shown^22,25,27,28^, EP2-R stimulation with PGE_2_ (10 μM) decreased the amplitude of glycine-evoked currents (Fig. 1A–C). The extent of inhibition was similar to what has previously been reported for dorsal horn neurons^22,25,27^. The application of PGE_2_ did not affect the glycine-activated currents in the absence of EP2-R expression (−6.5 ± 3.4%, P = 0.48, paired t-test). These results support that the stimulation of cAMP production triggers PKA-dependent phosphorylation of α3GlyRs. To study whether a GPCR-independent increase in cAMP levels may reduce the glycine-activated currents, we performed recordings in cells co-expressing α3GlyRs and a light-sensitive bacterial adenylyl cyclase, known as bPAC^30,31^. Activation of bPAC with blue light allows the optical triggering of cAMP production in living cells^30,31^. Our results showed that the activation of bPAC with blue light gradually diminished the glycine-activated current amplitudes in α3GlyRs by −35.5 ± 4.2% from control (P < 0.001, paired t-test). The degree of inhibition was not significantly different from that induced by EP2-R activation (P = 0.69, unpaired t-test) (Fig. 1A–C). Additional assays performed in cells expressing bPAC, but in the absence of light (Fig. 1A–C), showed stable currents over 10 minutes (−7.5 ± 5.3%, P = 0.19, paired t-test). Likewise, assays performed in cells expressing α3GlyRs recorded under blue light, but in the absence of bPAC (i.e. -bPAC + blue light, Fig. 1C), did not reveal significant current inhibition (−5.2 ± 3.9% from control, P = 0.25, paired t-test). Further experiments expressing α3GlyRs with a mutation in the consensus site for PKA-dependent phosphorylation (i.e. S346A) showed, in agreement with previous studies^22,27^, that the activation of bPAC did not significantly decrease the glycine-activated currents (−4.0 ± 8.8% from control, P = 0.67, paired t-test).

In order to assess whether the decrease in α3GlyR function triggered by PKA activation is associated with a loss of plasma membrane receptors, we studied cell-surface α3GlyRs under different conditions of PKA activation (Fig. 1D,E). Our results showed that the membrane expression of α3GlyRs after EP2-R stimulation or after bPAC activation was similar compared with control conditions (Fig. 1D,E). The quantitative analysis of the fluorescence intensity and number of receptor clusters on the cell membrane did not reveal significant differences (Fig. 1E). The analysis of additional conditions of PKA activation also showed a stable expression of α3GlyRs at the plasma membrane (See Supplementary Fig. 1).

### cAMP-mediated activation of PKA reduces the unitary conductance of α3GlyRs

**Figure 2:**
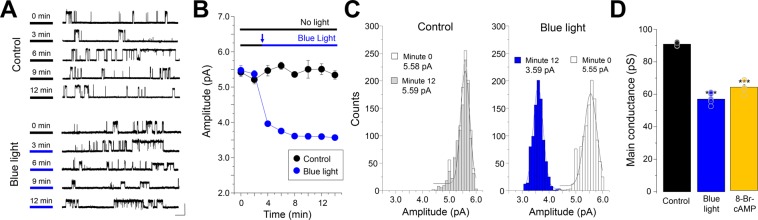
cAMP-mediated activation of PKA reduces the unitary conductance of wild-type α3GlyRs. (**A**) Single channel current traces obtained from cells co-expressing α3GlyRs and bPAC in the absence and presence of blue light. Calibration bar, 5 pA, 1 s. (**B**) Time course of the average amplitude of the events after blue light exposure (from minute 3) or in control conditions (12 minutes in no light condition). (**C**) The histograms show the amplitude distributions of control cells (left) and cells stimulated with blue light (right). (**D**) The plot summarizes the main conductance of α3GlyRs after blue light exposure or after the application of 8-Br-cAMP. Differences were significant (***P < 0.001; ANOVA followed by Bonferroni post-hoc test, F(2, 14) = 123.4). Control, n = 5; Blue light, n = 5; 8-Br-cAMP, n = 5.

In order to explore the impact of the activation of PKA signaling pathway on the elementary ion channel properties, we next performed single-channel recordings of α3GlyRs. In cells co-expressing bPAC and α3GlyRs, in the absence of light, the activation of α3GlyRs resulted in single-channel currents with an average amplitude of 5.47 ± 0.13 pA at +60 mV, as previously shown^27,32,33^. The single-channel amplitudes were stable during 12 minutes of recordings (P = 0.63, paired t-test) (Fig. 2A,B). Interestingly, we found that the activation of bPAC with blue light from the minute 3 of recording progressively diminished the unitary amplitude to 3.59 ± 0.05 pA (−34.4 ± 2.1%, blue light, minute 12, P < 0.001, paired t-test) (Fig. 2A,B). The comparison of the event distribution after 12 minutes confirmed that blue light exposure shifted the unitary currents towards lower amplitudes, whereas no changes in the amplitude distribution were detected in the absence of light (Fig. 2C). Additional calculations revealed a significantly lower conductance after blue light (control = 90.9 ± 0.6 pS vs blue light = 57.1 ± 2.3 pS, p < 0.001, unpaired t-test) (Fig. 2D). The α3GlyRs activity (measured as normalized open probability and mean open time) was not modified by the bPAC activation (Supplementary Table 1). In order to check whether a direct chemical stimulation of PKA could also modify the conductance, we next applied the non-hydrolyzable cAMP analog 8-Br-cAMP to cells expressing α3GlyRs. 8-Br-cAMP generated ion channels with a main conductance of 63.6 ± 2.01 pS, which were similar to the values obtained using bPAC (P = 0.17, unpaired t-test) (Fig. 2D).

### The residue S346 is an essential molecular determinant of the α3GlyR conductance

**Figure 3:**
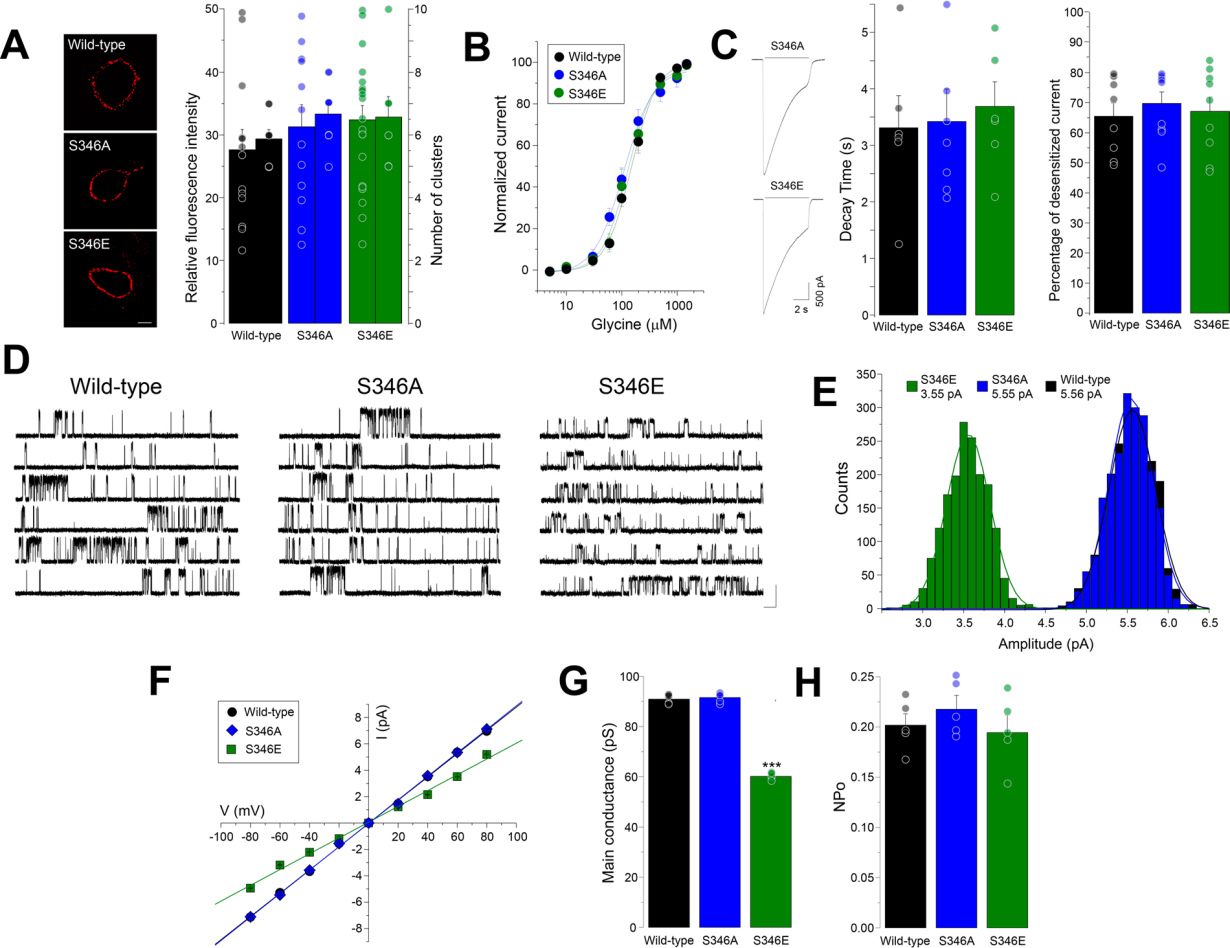
Characterization of α3GlyRs with mutations on the serine 346 residue. (**A**) Plasma membrane expression of wild-type, S346A and S346E α3GlyRs. The graph summarizes the average fluorescence intensity and the mean number of receptor clusters. Differences were not significant. (Fluorescence intensity, F(2, 45) = 0.73; number of clusters, F(2, 23) = 1.04). Calibration bar, 5 μm. Fluorescence intensity: Wild-type, n = 13; S346A, n = 12; S346E, n = 21; number of clusters**:** Wild-type, n = 8; S346A, n = 9; S346E, n = 7. (**B**,**C**) Concentration-response curves and glycine-activated currents from wild-type, S346A and S346E α3GlyRs. The mutations did not affect the glycine sensitivity nor the desensitization kinetics. Decay time: Wild-type, n = 6; S346A, n = 7; S346E, n = 6; Percentage of desensitized current**:** Wild-type, n = 8; S346A, n = 9; S346E, n = 9. (**D**) Single channel current traces obtained from cells expressing α3GlyRs or the mutated S346A or S346E constructs. Calibration bar, 5 pA, 1 s. (**E**,**F**) Single channel amplitude distributions (**D**) and current-voltage relationships (**F**) of wild-type, S346A and S346E α3GlyRs. (**G,H**) Average main conductance (**G**) and mean open probability (**H**) of wild-type α3GlyRs and the S346 mutated constructs. The unitary conductance was significantly reduced in the S346E mutant (***P < 0.001; ANOVA followed by Bonferroni post-hoc test, F(2, 14) = 732.6). The NPo was not significantly different (F(2, 14) = 0.67). Wild-type, n = 5; S346A, n = 5; S346E, n = 5.

Previous studies have shown that the conductance of pLGICs is determined primarily by the amino acid composition of the TM domains^4,34–36^. Nevertheless, other studies have shown that the mutation of charged residues in the ICD modified the conductance of pLGICs, including 5-HT3 receptors, nicotinic acetylcholine receptors and GlyRs^37–39^. The results described above suggest that the introduction of a single negative charge at position 346 of α3GlyRs should be able to reduce conductance. To explore this idea, we performed single-channel recordings of point-mutated α3GlyRs in which the key residue for PKA-dependent phosphorylation, serine 346, was mutated to a glutamate (S346E) or to an alanine (S346A)^27,40^ (Fig. 3). We first assessed the expression of these receptors in the plasma membrane. Our results showed that both mutated α3GlyRs displayed unaltered patterns of expression compared to wild-type (Fig. 3A). As previously reported^27,40^, our experiments showed that the apparent affinity for glycine of these GlyRs were not significantly modified by the mutations (EC_50_ wild-type = 141 ± 10 μM, n = 18; S346A = 106 ± 12 μM, n = 7; S346E = 128 ± 12 μM, n = 9; Fig. 3B). Analysis of the macroscopic currents indicated that the mutations did not significantly affect the decay time constant or the percentage of desensitized current (Fig. 3C). The single-channel analysis showed that the S346A mutant exhibited a mean amplitude of 5.49 ± 0.05 pA (Fig. 3D,E), which was not different from wild-type α3GlyRs (5.45 ± 0.04 pA, P = 0.51, unpaired t-test). Conversely, the average amplitude of the S346E mutant was significantly diminished to 3.58 ± 0.03 pA (Fig. 3D,E). The comparison of the event distribution confirms that the substitution of the S346 residue by glutamate shifted the unitary currents towards lower amplitudes, while the S346A construct was indistinguishable from wild-type receptors (Fig. 3E). Analysis of current-voltage relationships revealed that the mutations did not influence the rectification of the ion channel (Fig. 3F). Further calculations showed that the main conductance of the S346E construct was significantly reduced in comparison with the wild-type receptor or with the S346A mutant (Fig. 3G). However, the two mutated GlyRs did not present alterations on the normalized open probability or the mean open time (Fig. 3H, Supplementary Table 1).

### The role of the S346 residue on the ion channel conductance is conserved on a synthetic pLGIC

**Figure 4:**
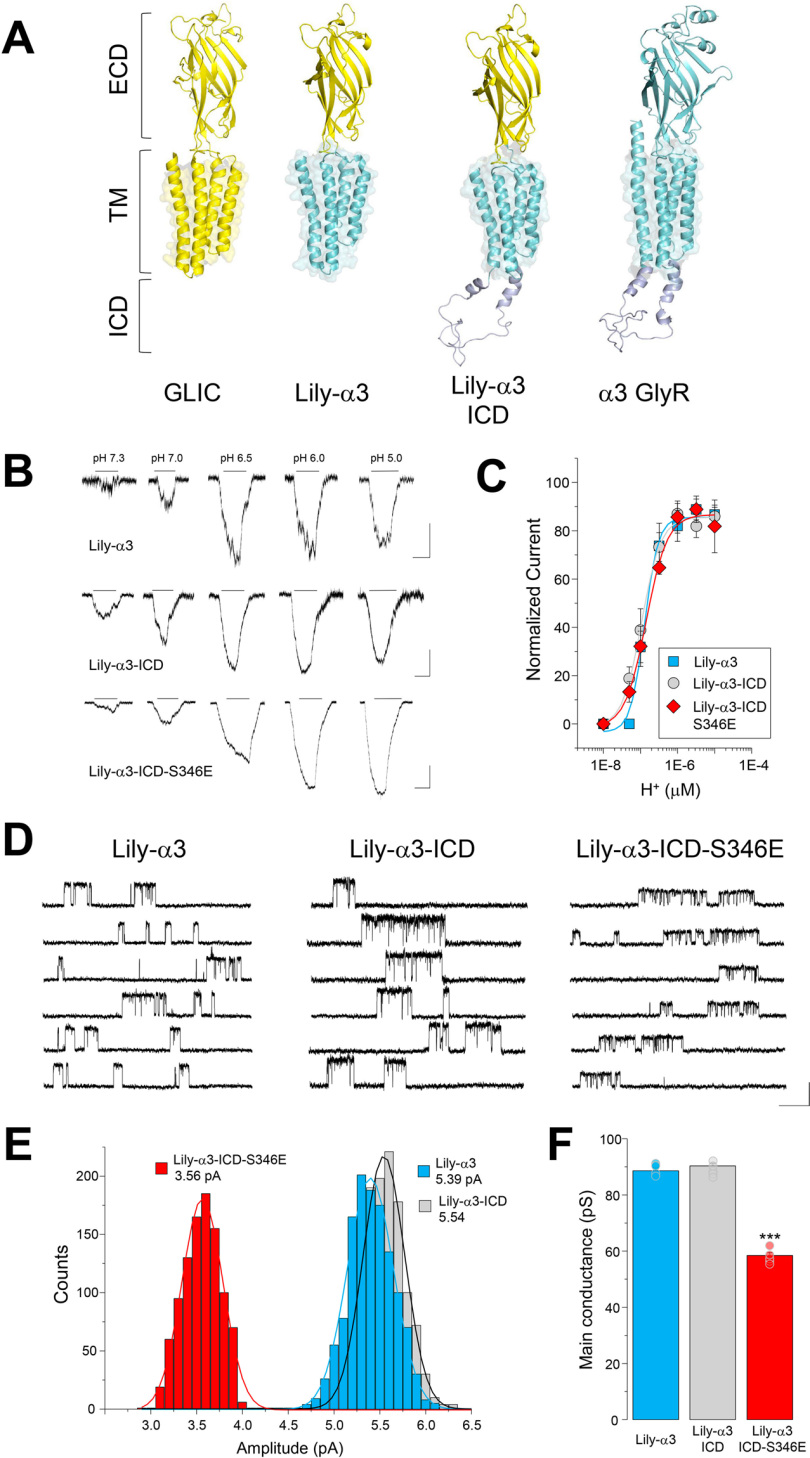
The unitary conductance of chimeric GLIC-α3GlyR receptors is determined by a single ICD residue. (**A**) The scheme summarizes the conformation of wild-type GLIC and α3GlyRs, together with the chimeric constructs Lily-α3 and Lily-α3-ICD. (**B**) The whole-cell current traces show proton-gated currents through Lily-α3, Lily-α3-ICD and Lily-α3-ICD-S346E receptor constructs. Calibration bar, 100 pA, 5 s. (**C**) Proton sensitivity of the Lily constructs expressed in BHK cells. Proton EC_50_, Lily-α3 = 1.3 × 10^−7^ ± 1.6 × 10^−8^ (pH = 6.9), (n = 7), Lily-α3-ICD = 1.1 × 10^−7^ ± 9.8 × 10^−9^ (pH = 6.9), (n = 12) and Lily-α3-ICD-S346E = 1.6 × 10^−7^ ± 5.9 × 10^−9^ (pH = 6.8), (n = 8). Differences were not significant. (**D**) Proton-gated unitary current traces obtained from cells expressing the respective Lily constructs. Calibration bar, 5 pA, 1 s. (**E**,**F**) Single channel amplitude distributions (**E**) and average conductance (**F**) of Lily-α3 (n = 5), Lily-α3-ICD (n = 5), and Lily-α3-ICD-S346E (n = 5) chimeric receptors. The unitary conductance was not influenced by the addition of the ICD but was diminished after the incorporation of a glutamate residue at position 346 (***P < 0.001; ANOVA followed by Bonferroni post-hoc test, F(2, 14) = 286.7).

A previous study postulated that the decrease of the glycine-activated currents through α3GlyRs elicited by S346 phosphorylation is due to structural changes within the glycine-binding site, which is located in the ECD^40^. Our results suggest that the reduction of the glycine-activated currents through α3GlyRs is mostly related to a decreased ion channel conductance, elicited by the chemical modification of S346 residue (Figs. 2–3) and thus likely restricted to the ICD. However, is currently unknown whether the presence of the ICD of α3GlyRs, as an independent module, is necessary and sufficient for the reduction of the single-channel conductance elicited by a change on the chemical composition of the S346 residue. In addition, the relevance of the ECD of α3GlyR for the reduction of the single-channel conductance reported here has been not determined. To test these questions, we employed a chimeric approach. Previous studies have shown that chimeric receptors combining the ECD of the bacterial GLIC channel and the TM domains of α1GlyRs (the so-called Lily channel) form proton-gated chloride-permeable ion channels^41,42^. Here, we designed and analyzed chimeric receptors composed of the ECD of the GLIC channel together with the TM and ICD domains of α3GlyRs (i.e. Lily-α3 constructs, Fig. 4A, see Supplementary Fig. 2). Whole-cell recordings showed the presence of proton-gated ion channels with similar agonist pharmacology (Fig. 4B,C). Interestingly, the single-channel analysis of the Lily-α3 receptor revealed an average amplitude of 5.31 ± 0.07 pA and a main conductance of 88.6 ± 1.18 pS, which resemble the values of wild-type α3GlyRs (Fig. 4D–F, see also Figs. 2–3). The incorporation of the α3GlyR-ICD to the Lily-α3 chimera (i.e. Lily-α3-ICD receptor) generated ion channels with a mean amplitude of 5.42 ± 0.06 pA and a unitary conductance of 90.3 ± 0.97 pS. These values were not different in comparison with the ICD-less counterpart (p = 0.26 and p = 0.30, unpaired t-test, Lily-α3 vs Lily-α3-ICD for amplitude and conductance, respectively). Nevertheless, the substitution of the S346 amino acid with a glutamate within the Lily-α3-ICD receptor generated ion channels with significantly decreased single-channel amplitudes (3.51 ± 0.06 pA, p < 0.001, unpaired t-test, Lily-α3-ICD vs S346E mutated construct) and diminished unitary conductance (58.5 ± 1.02 pS, p < 0.001, unpaired t-test, Lily-α3-ICD vs S346E mutated construct) (Fig. 4D–F). Further analyses showed that the normalized open probability and mean open time were not significantly different between these chimeric ion channels (Supplementary Table 1).

## Discussion

It is widely established that intracellular phosphorylation mediated by protein kinases is a relevant mechanism of modulation of pLGICs^4,43–49^. However, several aspects of the molecular, biophysical and structural mechanisms underlying the phosphorylation-dependent regulation of pLGIC function remain unclear. Our experiments showed that glycine-activated currents in α3GlyRs were diminished by both EP2-R activation or by a GPCR-independent increase in cAMP. At the biophysical level, diverse mechanisms may explain the decrease in ion channel amplitude. One possibility is the reduction of the ion channel expression at the cell surface. Previous reports have shown that phosphorylation by PKA or PKC results in the internalization of GlyRs^48,49^. Our results show that the plasma membrane expression of α3GlyRs remains stable after the activation of the cAMP-PKA pathway, suggesting that changes in the cell surface expression do not play a major role in the α3GlyRs inhibition. Another possibility involves changes in the affinity of the receptor to its agonist. A previous report suggest that the phosphorylation of residue S346 in α3GlyR alters the glycine-binding site structure^40^. Nevertheless, previous reports have shown that phospho-mimetic α3GlyRs (i.e. S346E mutant) displayed unaltered glycine sensitivity^27^. Thus, the reduction in the glycine-activated currents after PKA activation appears not clearly connected to changes in the agonist binding site.

The results of the present work support the idea that PKA-dependent inhibition of the α3GlyR function is associated to an alteration of the ion channel conductance. This concept is supported by at least three lines of evidence. First, PKA activation using bPAC reduced the unitary conductance of α3GlyRs. Second, a non-hydrolyzable cAMP analog diminished the conductance of α3GlyRs to a similar degree. Third, reduction in the conductance was mimicked by the substitution of the consensus site for PKA-dependent phosphorylation with a phospho-mimetic acidic residue. Interestingly, the S346E α3GlyR construct did not display any noticeable alteration in cell surface expression, open probability, desensitization, or rectification. Collectively, these results suggest that the functional inhibition of a pLGIC elicited by phosphorylation of a serine residue within the ICD is mostly associated with a reduction in the unitary conductance, rather than alterations in the cell surface receptors or agonist binding site.

pLGICs show a modular architecture in which each module (ECD, TMD and ICD) can be interchangeable between receptors to form functional chimeric channels^41,42,50,51^. We used this feature to address whether the substitution of a single residue within the ICD can affect the conductance of an engineered pLGIC and to determine the relevance of the ECD of α3GlyR for the conductance regulation by the ICD. The comparison of two chimeric receptors, one having the full ICD of α3GlyR (i.e. Lily-α3-ICD) and the other lacking an ICD (i.e. Lily-α3), showed similar proton sensitivities, analogous unitary conductances and comparable open probability. These observations suggest that inclusion of the ICD does not produce striking changes in essential ion channel features. Interestingly, this idea is supported by other reports showing that ICD insertion or replacement between eukaryotic pLGICs and bacterial GLIC channels did not produce alterations in the conductance or ion selectivity^52,53^.

From an evolutionary point of view, these observations are interesting because the overall architecture and functional features of pLGICs are conserved from archaea to metazoan^54,55^. Therefore, since the ICD only emerged later in the evolution of eukaryotic pLGICs, one might argue that the ICD has been included merely an accessory domain. Our results showed however that introduction of the S346E mutation into the ICD of the Lily-α3-ICD chimera and of the wild-type α3GlyR produced a significant reduction in the unitary conductance. Together with the results that show a decrease in the conductance after PKA activation, our findings suggest that the phosphorylation state of the S346 residue determines the unitary conductance of α3GlyRs. In addition, our data using chimeric channels showed that the ICD-dependent reduction of the conductance did not requires a glycine-binding site within the ECD. Taken together, our evidences suggest that the contribution of the ICD to the conductance of α3GlyRs appears to be dependent on the amino acid composition of this particular region, rather than on its presence. In a wider context, these results suggest that reversible post-translational modifications targeting a single residue within the ICD of a pLGIC may change the channel conductance, strengthen the idea that the ICD, likely as an independent module, is an important determinant of intrinsic pLGICs properties^37–39,56–58^.

The function of GlyRs has been traditionally linked to neuronal inhibition at the level of spinal cord^2,3,47^. These studies have determined that α3-containing GlyRs are expressed in superficial layers of the dorsal horn, contributing to nociceptive processing and chronic pain of inflammatory origin^2,22^. An increasing number of studies have suggested in addition that GlyRs are also expressed in supraspinal sites^5,6,9–11,59,60^. Interestingly, recent immunocytochemical and gene expression studies have determined that α3GlyRs are expressed in brainstem, trigeminal ganglia, nucleus accumbens and the hippocampus^17,20,21,23,59–61^. Despite the absence of specific pharmacological tools to identify α3GlyRs, the presence of functional α3-containing GlyRs in several CNS areas has been inferred by using mice lacking α3GlyRs. These studies have found that α3GlyRs participate in both synaptic and tonic glycinergic inhibition^11,22,23,27^. The unitary conductance regulation of α3GlyRs by the activation of PKA signaling pathway described here provides a plausible regulatory mechanism of glycine-activated chloride influx in these CNS regions. Based on these results, we speculate that impaired chloride conductance may underlie, at least in part, dynamic glycinergic dis-inhibition associated with phosphorylated α3GlyRs, such as inflammatory pain sensitization in the spinal cord and respiratory control by the brainstem^22,23^. Phosphorylation may also contribute to the regulation of tonic inhibition strength associated with α3GlyRs in forebrain structures^11^. Although the present work defined a possible mechanism of glycinergic dysfunction at the biophysical level, whether this phenomenon contributes to the modulation of native GlyRs is still a scientific challenge due to the absence of GlyR subunit-specific inhibitors to study pharmacologically-isolated α3GlyRs in a neuronal context. Further experiments will define whether this regulatory mechanism of α3GlyRs contributes to the control of neuronal activity.

In conclusion, we demonstrate that the activation of PKA signaling pathway decreases the unitary conductance of α3GlyRs. Our observations point out the importance of the chemical composition of the ICD as a critical determinant for the pLGIC conductance and provide a plausible biophysical mechanism to explain processes of glycinergic dis-inhibition. Although further studies are necessary to understand the structural changes involved, these results may open further possibilities for the design and development of new compounds targeting phosphorylated α3GlyRs, which may recover receptors with unpaired conductance and may have interesting clinical applications.

## Methods

### Expression plasmids, cell culture, and transfection

The α3L version of the α3GlyR was used in our studies. Mutations were inserted by using the Quick-Change site-directed mutagenesis kit (Agilent). Chimeric receptors composed of the bacterial GLIC channel and α3GlyRs were designed based on previous protocols^41,42^. The chimeras were then chemically synthesized (General Biosystems, USA) and subcloned in a pCDNA3 vector (Invitrogen). The cells were transfected using XfectTM Transfection Reagent (Clontech, San Francisco, CA, USA) with 0.5–3.0 μg of cDNA plasmids encoding ion channels and 0.5 μg of EGFP. HEK293 cells were used unless otherwise stated. To avoid endogenous proton-gated currents, BHK cells were used in the experiments involving GLIC-GlyR chimeras. In the experiments involving the activation of bPAC or EP2 receptors, 2 μg of each plasmid were used. The photosensitive bacterial adenylyl cyclase (bPAC) was a gift from Dr. Peter Hegemann (Addgene plasmid # 28134)^30^. The experiments involving optical stimulation of bPAC were performed using mCherry as a transfection marker. The recordings were made 36–48 hours after transfection.

### Chemicals

PGE2 was purchased from Tocris (Bristol, UK). All other reagents were from Sigma-Aldrich (St. Louis, MO, USA).

### Electrophysiology

The glycine-activated currents were recorded from transiently transfected HEK 293 cells in the whole-cell voltage-clamp configuration (−60 mV). The recordings were performed at room temperature (20–24 °C)^27,41,42^. The patch electrodes (3–4 mΩ) were filled with an intracellular solution containing (in mM): 120 CsCl, 8 EGTA, 10 HEPES (pH 7.4), 4 MgCl2, 0.5 GTP and 2 ATP. The bath solution contained (in mM) 140 NaCl, 5.4 KCl, 2.0 CaCl2, 1.0 MgCl2, 10 HEPES (pH 7.4), and 10 glucose. The recordings were performed with an Axoclamp 200B amplifier (Molecular Devices, USA) or with a HEKA EPC10 (HEKA Elektronik GmbH, Germany). The signals were acquired using Clampex 10.1 or PatchMaster software. Data analysis was performed off-line using Clampfit 10.1 (Axon Instruments, Sunnyvale, CA, USA). The exogenous glycine-evoked currents were obtained using an outlet tube (200 μm ID) of a custom-designed gravity-fed microperfusion system, positioned 50–100 μm from the recorded cell. The agonist was manually applied to the cell using a short pulse (3–4 s). Concentration-response curves were obtained from normalized concentration–response data points. The optical stimulation of bPAC was performed using a fiber-coupled LED emitting blue light (470 nm) and a T-Cube LED Driver (Thorlabs, USA). Glycine stocks were prepared daily in high purity distilled water. The stock solutions of PGE2 were prepared in DMSO and kept at −20 °C. Single channel recordings were performed as previously reported^32,33,42^. The recordings were performed in the cell-attached configuration (+60 mV). The patch pipettes (10–20 mΩ) were manually fire-polished in a microforge (Narishige, Japan). The extracellular solution contained (in mM): 20 Na-gluconate, 102.7 NaCl, 2 KCl, 2 CaCl2, 1.2 MgCl2, 10 HEPES, 20 TEA-Cl, 15 sucrose, and 14 glucose, pH 7.4. The pipette was filled with extracellular solution containing glycine (100–200 μM). 8-Br-cAMP (100 μM) was applied to the extracellular solution 15 minutes prior to the recordings. The data were filtered (2-kHz low-pass 8-pole Butterworth) and acquired at 5 kHz using an Axopatch 200B amplifier and a 1322 A Digidata (Axon Instruments, Union City, CA). Data was acquired using pClamp software and analyzed off-line with Clampfit 10.1 (Axon Instruments, Union City, CA). Single-channel conductance (γ) values were determined from the relationship γ = I/(V_m_ − V_rev_), in which I is the current amplitude of single channel events, V_m_ is the membrane potential and Vrev the reversal potential.

### Immunocytochemistry

To analyze cell surface GlyRs, we used a C-terminal hexa-histidine tagged α3GlyR version. Transfected cells were first washed 3 times with PBS and then incubated with a monoclonal hexa-histidine antibody (1:400 anti-6xHis, Clontech) for 10 minutes at 37 °C and 5% CO_2_. Subsequently, the cells were fixed with 4% paraformaldehyde (0.1 M phosphate buffer, pH 7.4) for 15 minutes at 4 °C and blocked with 10% horse serum for 5 min. Epitope visualization was performed by incubating the sample with a secondary antibody Cy3 (1:200; Jackson ImmunoResearch Laboratories, USA). Finally, the cells were cover-slipped with Fluorescence Mounting Medium (Dako, CA, USA). For quantitative analysis, cells were randomly chosen for imaging using a confocal microscopy (Zeiss LSM700 spectral confocal microscope). Single stacks of optical sections in the z-axis (1 µm) were acquired, and dual-color immunofluorescent images were captured in simultaneous two-channel mode. The quantification of the fluorescence intensity and number of clusters associated with membrane receptors was carried out off-line using ImageJ software (NIH, Bethesda, MD, USA), using previously reported plug-ins and protocols^62^. Briefly, for each cell analyzed, the relative fluorescence units (URF) of the red channel were obtained by averaging 3 zones with areas of 100 μm^2^. For the quantification of clusters, the stacks obtained on the Z axis were first placed in a 25 μm × 4 μm box, and then the number of distinguishable points were quantified.

### Molecular modeling

The full model of the α3GlyR was created using the structure of the human α3GlyR [PDB ID: 5 TIO] and the ICD predicted ab initio with QUARK, as previously described^51^. The models of Lily-α3 and Lily-α3-ICD subunits were constructed by homology modeling using the structure of the chimera GLIC-α1GlyR called “Lily” [PDB ID: 4 × 5T] as template^42^. For both chimeric receptors, the section corresponding to α1GlyR in “Lily” was replaced with the sequence of the human α3GlyR from the amino acid 218 (in TM1) to the C-terminal end. In the Lily-α3 model, the ICD was replaced with the sequence SQP^42^ (See Supplementary Fig. 2). To generate Lily-α3-ICD, the model of the α3-ICD, was first generated using Modeller as previously described^51^ and subsequently added to Lily-α3 model. All final models were obtained after filling missing side chains with Prime (version 2016-2, Schrödinger, LLC, New York, NY, 2016) and energy minimization with a conjugate gradient protocol in the software MacroModel (version 2016-2, Schrödinger, LLC, New York, NY, 2016). All images were created with PyMOL (version 1.5, DeLano Scientific LLC)^27,51^.

### Data analysis

All values were expressed as mean ± SEM. Statistical comparisons were performed using Student t-tests. Multiple comparisons were analyzed with ANOVA followed by a Bonferroni post hoc test. P values less than 0.05 were considered statistically significant. All the statistical analyses and plots were performed with MicroCal Origin 6.0 or 8.0 (Northampton, MA, USA).

## Supplementary information

Supplementary Information.
