# Supplementary material for: Modulation of glycine receptor single-channel conductance by intracellular phosphorylation
Source: Sci Rep. 2020 Mar 16;10:4804. doi: 10.1038/s41598-020-61677-w (PMC7076024; doi:10.1038/s41598-020-61677-w)
Supplement: Supplementary file 1 — Supplementary Information. [file 41598_2020_61677_MOESM1_ESM.pdf]

Supplementary Information

"Modulation of glycine receptor single-channel conductance by intracellular phosphorylation"

Gustavo Moraga-Cid[a,1,*], Victoria P. San Martín[a,1], Cesar O. Lara[a], Braulio Muñoz[b], Ana M. Marileo[a], Anggelo Sazo[a], Carola Muñoz-Montesino[a], Jorge Fuentealba[a], Patricio A. Castro[a], Leonardo Guzmán[a], Carlos F. Burgos[a], Hanns U. Zeilhofer[c,d], Luis G. Aguayo[a], Pierre-Jean Corringer[e] , Gonzalo E. Yévenes[a,*]

[1] These authors contributed equally to this work

[*] To whom correspondence may be addressed:

Dr. Gustavo Moraga-Cid, Department of Physiology, Faculty of Biological Sciences, University of Concepcion, Chile. gumoraga@udec.cl

Dr. Gonzalo E. Yévenes, Department of Physiology, Faculty of Biological Sciences, University of Concepcion, Chile. gyevenes@udec.cl

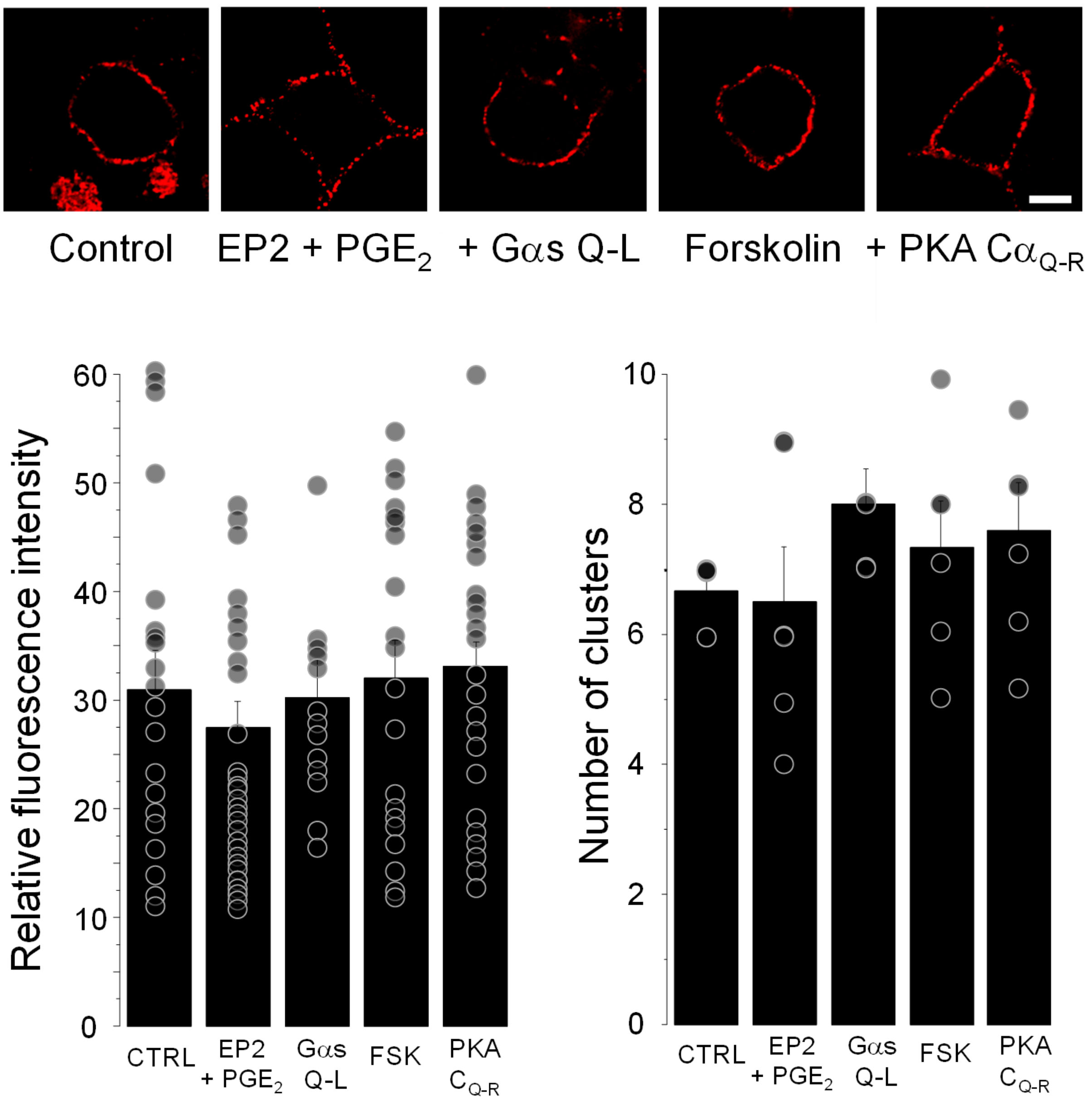

Supplementary figure 1. Plasma membrane expression of α3GlyRs after the activation of the cAMP-PKA pathway. A. Representative Images of HEK293 cells expressing α3GlyRs together with EP2 receptors, with a constitutively active Gαs (i.e. Gαs Q-L) or with a mutated active PKA Cα subunit (PKA CαQ-R). Cells incubated with the adenylyl cyclase activator forskolin (10 μM, 30 min) are also shown. Vehicle condition corresponds to an incubation (30 min) of the cells with 0.4% DMSO. The Gαs Q-L and PKA CαQ-R expression plasmids were previously described (8-10). Calibration bar, 5 μm. **B**. The graphs show the quantification of the receptor-associated signals (left) and the number of receptor clusters (right). Differences were not significant. Receptor-associated signals: control, n=20; EP2+PGE2, n=33; Gαs Q-L, n=16; FSK, n=23; PKA CαQ-R, n=30. Number of clusters: control, n=6; EP2+PGE2, n=6; Gαs Q-L, n=7; FSK, n=7; PKA CαQ-R, n=6.

```
GLIC          QDMVSPPPPIADEPLTVNTGIYL-IECYSLDDKAET---FKVNAFLSLSWKDRRLAFDPVRSGVRV-KTYEPEAIWIPEIRFVNVENARDADVVD
α3GlyR        GYDARIRPNFK-GP-PVNVTCNIFI—NSFGSIAETTMDYRVNIFLRQKWNDPRLAYSEYPDDSLDLDPSMLDSIWKPDLFFANEKGANFHEVTTD
Lily-α3-ICD   QDMVSPPPPIADEPLTVNTGIYL-IECYSLDDKAET---FKVNAFLSLSWKDRRLAFDPVRSGVRV-KTYEPEAIWIPEIRFVNVENARDADVVD
Lily-α3       QDMVSPPPPIADEPLTVNTGIYL-IECYSLDDKAET---FKVNAFLSLSWKDRRLAFDPVRSGVRV-KTYEPEAIWIPEIRFVNVENARDADVVD
Lily          QDMVSPPPPIADEPLTVNTGIYL-IECYSLDDKAET---FKVNAFLSLSWKDRRLAFDPVRSGVRV-KTYEPEAIWIPEIRFVNVENARDADVVD

GLIC          -ISVSP--DGTVQYLERFSARVLS-PLDFRRYPFDSQTLHIYLIVRSVDTRNIVLAVDLEKVGKNDDVFLTGWDIESFTAVVKP---ANFALEDRL
α3GlyR        NKLLRIFKNGNVLYSIRLTLT-LSCPMDLKNFPMDVQTCIMQLESFGYTMNDLIFEWQDEAPVQVAE-GLT--LPQFLLKEEKDLRYCTKHYNTGK
Lily-α3-ICD   -ISVSP--DGTVQYLERFSARVLS-PLDFRRYPFDSQTLHIYLIVRSVDTRNIVLAVDLEKVGKNDDVFLTGWDIESFTAVVKP---ANFALEDRL
Lily-α3       -ISVSP--DGTVQYLERFSARVLS-PLDFRRYPFDSQTLHIYLIVRSVDTRNIVLAVDLEKVGKNDDVFLTGWDIESFTAVVKP---ANFALEDRL
Lily          -ISVSP--DGTVQYLERFSARVLS-PLDFRRYPFDSQTLHIYLIVRSVDTRNIVLAVDLEKVGKNDDVFLTGWDIESFTAVVKP---ANFALEDRL

                                    TM1                        TM2                           TM3
GLIC          ESKLDYQLRISRQYFSYIPNIILPMLFILFISWTAFWST--SYEANVTLVVSTLIAHIAFNILVETNLPKTPYMTYTGAIIFMIYLFYFVAVIEVT
α3GlyR        FTCIEVRFHLERQMGYYLIQMYIPSLLIVILSWVSFWINMDAAPARVALGITTVLTMTTQSSGSRASLPKVSYVKAIDIWMAVCLLFVFSALLEYA
Lily-α3-ICD   ESKLDYQLRISRQMGYYLIQMYIPSLLIVILSWVSFWINMDAAPARVALGITTVLTMTTQSSGSRASLPKVSYVKAIDIWMAVCLLFVFSALLEYA
Lily-α3       ESKLDYQLRISRQMGYYLIQMYIPSLLIVILSWVSFWINMDAAPARVALGITTVLTMTTQSSGSRASLPKVSYVKAIDIWMAVCLLFVFSALLEYA
Lily          ESKLDYQLRISRQMGYYLIQMYIPSLLIVILSWISFWINMDAAPARVGLGITTVLTMTTQSSGSRASLPKVSYVKAIDIWMAVCLLFVFSALLEYA

GLIC          VQHYLKVE----------------------------SQP-----------------------------------------------------ARA
α3GlyR        AVNFVSRQHKELLRFRRKRKNKTEAFALEKFYRFSDTDDEVRESRFSFTAYGMGP-CLQAKDGVVPKGPNHAVQVMPKSAD-----EMRKVFIDRA
Lily-α3-ICD   AVNFVSRQHKELLRFRRKRKNKTEAFALEKFYRFSDTDDEVRESRFSFTAYGMGP-CLQAKDGVVPKGPNHAVQVMPKSAD-----EMRKVFIDRA
Lily-α3       AVNFVSRQ----------------------------SQP-----------------------------------------------------DRA
Lily          AVNFVSRQ----------------------------SQP-----------------------------------------------------QRA

                       TM4
GLIC          ASITRASRIAFPVVFLLANIILAFLFFGF
α3GlyR        KKIDTISRACFPLAFLIFNIFYWVIYKILRHEDIHHQQD
Lily-α3-ICD   KKIDTISRACFPLAFLIFNIFYWVIYKILRHEDIHHQQD
Lily-α3       KKIDTISRACFPLAFLIFNIFYWVIYKILRHEDIHHQQD
Lily          KKIDKISRIGFPMAFLIFNMFYWIIYKIVRREDVHNQ
```

Supplementary figure 2. Primary sequence of GLIC-α3GlyR chimeric receptors. A. The alignment shows the detailed composition of the chimeric receptors composed by modules of the bacterial GLIC channel and the α3GlyR. These constructions were design based on the Lily receptor composed by GLIC and the α1GlyR. The S346 residue of α3GlyR is highlighted in red on the Lily-α3-ICD sequence.

α3GlyR + bPAC			
**Condition**	**NPo**	**MOT (ms)**	**n**
Control	0.26 ± 0.02	380 ± 98	5
Blue Light	0.23 ± 0.03	346 ± 76	5
**a3GlyR, wild-type, S346A, S346E**			
**Wild Type**	0.20 ± 0.02	321 ± 72	5
S346A	0.21 ± 0.03	286 ± 56	5
S346E	0.19 ± 0.03	399 ± 61	5
**Chimeric GlyRs**			
**Lily-α3**	0.28 ± 0.03	396 ± 89	5
Lily-α3-ICD	0.23 ± 0.02	355 ± 76	5
Lily-α3-ICD-S346E	0.25 ± 0.02	389 ± 58	5

Supplementary Table 1. Single-channel kinetic parameters. The data reveal that none of the conditions significantly modified the nPo (normalized open probability) or the MOT (mean open time). These results suggest that the PKA-activation nor the mutations in the S346 residue affected the ion channel activity.
